# Integrated Transcriptional Profiling Analysis and Immune-Related Risk Model Construction for Intracranial Aneurysm Rupture

**DOI:** 10.3389/fnins.2021.613329

**Published:** 2021-04-01

**Authors:** Dezhi Shan, Xing Guo, Guozheng Yang, Zheng He, Rongrong Zhao, Hao Xue, Gang Li

**Affiliations:** ^1^Department of Neurosurgery, Qilu Hospital, Cheeloo College of Medicine and Institute of Brain and Brain-Inspired Science, Shandong University, Jinan, China; ^2^Shandong Key Laboratory of Brain Function Remodeling, Jinan, China; ^3^Department of Neurosurgery, Qilu Hospital (Qingdao), Cheeloo College of Medicine, Shandong University, Qingdao, China

**Keywords:** intracranial aneurysms, functional enrichment analysis, immune cell infiltration, immune-related risk model, ceRNA network

## Abstract

Intracranial aneurysms (IAs) may cause lethal subarachnoid hemorrhage upon rupture, but the molecular mechanisms are poorly understood. The aims of this study were to analyze the transcriptional profiles to explore the functions and regulatory networks of differentially expressed genes (DEGs) in IA rupture by bioinformatics methods and to identify the underlying mechanisms. In this study, 1,471 DEGs were obtained, of which 619 were upregulated and 852 were downregulated. Gene enrichment analysis showed that the DEGs were mainly enriched in the inflammatory response, immune response, neutrophil chemotaxis, and macrophage differentiation. Related pathways include the regulation of actin cytoskeleton, leukocyte transendothelial migration, nuclear factor κB signaling pathway, Toll-like receptor signaling pathway, tumor necrosis factor signaling pathway, and chemokine signaling pathway. The enrichment analysis of 20 hub genes, subnetworks, and significant enrichment modules of weighted gene coexpression network analysis showed that the inflammatory response and immune response had a causal relationship with the rupture of unruptured IAs (UIAs). Next, the CIBERSORT method was used to analyze immune cell infiltration into ruptured IAs (RIAs) and UIAs. Macrophage infiltration into RIAs increased significantly compared with that into UIAs. The result of principal component analysis revealed that there was a difference between RIAs and UIAs in immune cell infiltration. A 4-gene immune-related risk model for IA rupture (IRMIR), containing CXCR4, CXCL3, CX3CL1, and CXCL16, was established using the glmnet package in R software. The receiver operating characteristic value revealed that the model represented an excellent clinical situation for potential application. Enzyme-linked immunosorbent assay was performed and showed that the concentrations of CXCR4 and CXCL3 in serum from RIA patients were significantly higher than those in serum from UIA patients. Finally, a competing endogenous RNA network was constructed to provide a potential explanation for the mechanism of immune cell infiltration into IAs. Our findings highlighted the importance of immune cell infiltration into RIAs, providing a direction for further research.

## Introduction

Intracranial aneurysms (IAs) are abnormal bumps that occur in an arterial wall, and they are an important cause of spontaneous subarachnoid hemorrhage (SAH) (Macdonald and Schweizer, 2017). In the past few years, high blood pressure, smoking, atherosclerosis, and other factors have been shown to cause the occurrence and development of IAs (Bakker et al., 2020; Ogilvy et al., 2020). However, the molecular mechanisms of the formation and rupture of IAs remain unclear. With the rapid development of microarray technology, the GEO database has gradually played an important role in bioinformatics analysis (Zhang et al., 2019). In this study, we used bioinformatics analysis to discover differentially expressed genes (DEGs) between ruptured IAs (RIAs) and unruptured IAs (UIAs), and we constructed a protein–protein interaction (PPI) network and searched for relationships. Inflammation and immune cell infiltration, including macrophages, mast cells (Furukawa et al., 2020), and monocytes, play important roles in the formation and rupture of IAs. A previous study confirmed that inflammation-accelerated Toll-like receptor 4 (TLR4) pathway in aneurysmal walls promoted the development of IA rupture (Mitsui et al., 2020). Similarly, Kanematsu et al. (2011) reported that the infiltration and polarization of macrophages were closely related to the rupture of IAs.

Intriguingly, cytokines and collagenases secreted by macrophages in inflammation participated in the progress of rupture of IAs. Monocyte chemoattractant protein-1 (MCP-1) secreted by macrophages may play a critical role in aneurysm development as a major chemoattractant for monocytes/macrophages, which are induced through nuclear factor κB (NF-κB) activation (Aoki et al., 2009). The tumor necrosis factor α (TNF-α)/NF-κB pathway may be of great importance in the development of IAs (Aoki et al., 2014). TNF-α stimulates endothelial cells, fibroblasts, and smooth muscle cells, which upregulate adhesion molecules that act as cytokines to attract leukocytes to facilitate the migration of recruited leukocytes (Borish and Steinke, 2003). We used CIBERSORT, which is a bioinformatics method, to analyze the infiltration of immune cells by deconvolution of transcriptional profiles (Newman et al., 2015). An immune-related risk model for IA rupture (IRMIR) based on immune-related hub genes was explored. Enzyme-linked immunosorbent assay (ELISA) was used to quantitatively determine the concentration of IRMIR genes in serum.

Long noncoding RNAs (lncRNAs) are RNAs of more than 200 nucleotides in length (Ma et al., 2020), and they have direct and indirect regulatory roles in tumors, cardiovascular diseases, and other diseases (Luo et al., 2019; Omura et al., 2020). More recently, some studies have explored the lncRNA expression profiles in IAs (Sun et al., 2020; Tutino et al., 2020). It is widely accepted that lncRNAs function by regulating the binding of microRNAs to their target mRNAs, which is known as the competitive endogenous RNA (ceRNA) hypothesis. Few studies on the lncRNA–miRNA–mRNA ceRNA networks between RIAs and UIAs have been conducted. In this study, ceRNA networks were constructed to provide a potential explanation for inflammation and immune cell infiltration in IAs.

## Materials and Methods

### Data Downloading and Processing

Microarray data, containing five transcription profiles (GSE13353, GSE26969, GSE6551, GSE54083, and GSE15629) were downloaded from the NCBI GEO database^1^. The first three transcription profiles are all based on the GPL570 platform. GSE13353 contains 8 UIA samples and 11 RIA samples. GSE26969 includes three UIA samples and three normal superficial temporal artery (STA) samples, and three UIA samples were selected. GSE6551 contains three UIA samples, 2 RIA samples and five STA samples, and two RIA samples were selected. Thus, a gene expression matrix including 11 UIA samples and 13 RIA samples was obtained for DEG analysis. GSE54083 includes 5 UIA, 8 RIA, and 10 control STA samples, and 5 UIA and 8 RIA samples were selected for the subsequent IRMIR construction. GSE15629 includes six UIA, eight RIA, and five control middle meningeal artery samples, and six UIA and eight RIA samples were selected for the subsequent IRMIR construction. For the downloaded microarray data, raw sequence data were processed using Perl scripts^2^, and batch normalization between groups was performed. A gene annotation file (GRCh38) downloaded from the Ensembl genome browser^3^ was used to annotate the genes encoding proteins and lncRNAs on the corrected transcription profiles.

### DEG and Functional Enrichment Analyses

The expression data from the microarray assay were processed by the limma package^4^, and the genes with an adjusted *p* value of less than 0.05 and a log_2_| (fold change)| value greater than 1 were considered to be differentially expressed between the RIA and UIA samples. The pheatmap package was used to conduct hierarchical clustering for DEGs in R. Gene Ontology (GO) functional enrichment and Kyoto Encyclopedia of Genes and Genomes (KEGG) pathway analyses of the DEGs were performed using the DAVID online tool^5^. Gene set enrichment analysis (GSEA) was performed using GSEA software^6^.

### Construction of a Protein–Protein Interaction Network and Subnetwork and Identification of Hub Genes

Based on the STRING database^7^, the DEGs were used to construct a PPI network, and the network node files were downloaded and exported to Cytoscape software for visualization. The MCODE plug-in in Cytoscape was used to construct the subnetwork, and the Degree Cutoff and K-Core were both set to 7. Using the cytoHubba plug-in in Cytoscape, the top 20 genes calculated by the Multiscale Curvature Classification (MCC) algorithm were selected as hub genes. Metascape (Zhou et al., 2019b) was used to perform enrichment analysis and visualization of the subnetwork and hub genes.

### Weighted Gene Coexpression Network Analysis of DEGs

The WGCNA package was used for weighted gene coexpression network analysis (WGCNA) in R. Expression profile data for the DEGs were used in the WGCNA, and the modules with obvious clustering were utilized for functional enrichment analysis. We selected a power of β = 16 and a scale-free *R*^2^ = 0.86 as the soft-thresholding parameters to ensure an assigned scale-free coexpression gene network.

### Immune Cell Infiltration Analysis of RIAs and UIAs

Using expression data from the microarray assay, the immune cell infiltration analysis of RIAs and UIAs was performed by CIBERSORT to predict the compositions and contents of immune cells. Heatmaps and histograms were generated to show the compositions of immune cells in different samples using the pheatmap package in R. A correlation heatmap was generated to show the correlation of immune cells using the corrplot package in R. A violin map was graphed in R to show the differences in immune cells between the RIA and UIA groups using the vioplot package. Principal component analysis (PCA) of immune cell infiltration between RIAs and UIAs was performed by the ggplot2 package.

### Construction of an Immune-Related Risk Model of IA Rupture

Immune-related genes were obtained from the Immport website^8^. The expression profiling data for the intersections of hub genes and immune-related genes were used as a training set for construction of the IRMIR. Corresponding genes in the GSE54083 and GSE15629 data series were used as test set 1 and test set 2 in the model construction, respectively. The glmnet package in R software and logistic regression were used to construct the IRMIR. The PROC package was used to draw the receiver operating characteristic (ROC) curves.

### Biological Sample Collection

In total, this study recruited 10 participants (aged 50–77 years), including five UIA patients (mean age of 62.6 years) and five RIA patients (mean age of 60.6 years) from the Neurosurgery Department in Qilu Hospital of Shandong University. All the patients met the diagnostic criteria of IA guidelines. The patients were diagnosed by digital subtraction angiography, computed tomography angiography, or magnetic resonance angiography, and they were confirmed by two doctors in relevant departments.

The patients were all diagnosed for the first time without surgery or drug treatment. Patients were excluded if any of the following conditions were met: (1) treated with aneurysm clipping or intravascular therapy before admission; (2) history of autoimmune disease, peripheral vascular disease, or cancer; (3) severe cardiac, hepatic, and renal insufficiency; (4) pregnant or lactating women; (5) mental disorders that prevented cooperation with the test. The Research Ethics Committee of Qilu Hospital of Shandong University approved the research proposal. We obtained written informed consent from the participants at enrollment. Blood (5 mL) was collected by venipuncture in vacuum tubes containing heparin on the same day of the clinical assessment. Whole blood samples were used for plasma within 2 h of collection. Blood samples were centrifuged twice at 3,000 g for 10 min at 4°C. Serum was collected and stored at −80°C.

### ELISA and Statistical Analysis

Samples were thawed, and serum levels of CXCR4 and CXCL3 were measured by ELISA according to the manufacturer’s protocol (CUSABIO, China). CXCR4 (CSB-E12825h) and CXCL3 (CSB-EL006249HU) assays employed the quantitative sandwich ELISA technique. Briefly, for the sandwich ELISA–based assays (CXCR4 and CXCL3), standards and samples were pipetted into the wells of a plate that had been precoated with antibodies specific for each marker to be analyzed. Any CXCR4/CXCL3 present was bound by the immobilized antibody. After removing any unbound substances, a biotin-conjugated antibody specific for CXCR4/CXCL3 was added to the wells. After washing, avidin-conjugated horseradish peroxidase was added to the wells. Following a wash to remove any unbound avidin-enzyme reagent, a substrate solution (3,3t,5,5t-tetramethylbenzidine, TMB) was added to the wells, and the color developed in proportion to the amount of CXCR4/CXCL3 bound in the initial step. The color development was stopped by adding sulfuric acid solution, and the intensity of the color was measured spectrophotometrically at a wavelength of 450 nm.

The concentrations were calculated based on a standard curve in which the absorbance was plotted against the standard concentration. The sensitivity of the assays was 3.0 pg/mL for CXCR4 and CXCL3. All variables were tested for Gaussian distribution by the Shapiro–Wilk normality test. Independent-sample *t* test was used to determine significant differences between pairs of groups. All statistical tests were two-tailed and were performed using a significance level of α = 0.05. Statistical analyses were performed using GraphPad Prism version 8.0 (GraphPad Software, Inc., La Jolla, CA, United States).

### Construction of Immune-Related Competitive Endogenous RNA Networks

The limma package was used to perform differential analysis based on the threshold (*p* < 0.05) and log_2_| (fold change)| values > 1 to obtain differentially expressed lncRNAs (DElncRNAs), and the pheatmap package was used to conduct hierarchical clustering for the DElncRNAs in R. The missing expression value was replaced by the mean value of each group. Perl script was used to predict the differentially expressed microRNAs (DEmiRNAs) corresponding to the DElncRNAs. The intersection of DEGs and immune-related genes were considered immune-related DEGs (IDEGs). The target genes of the DEmiRNAs were predicted using the miRDB, miRTarBase, and TargetScan databases, and the intersection of IDEGs and the predicted results were considered the differentially expressed mRNAs (DEmRNAs). The resulting interactions were used to construct the lncRNA-miRNA-mRNA network using Cytoscape software.

## Results

### DEG and Functional Enrichment Analyses

**FIGURE 1:**
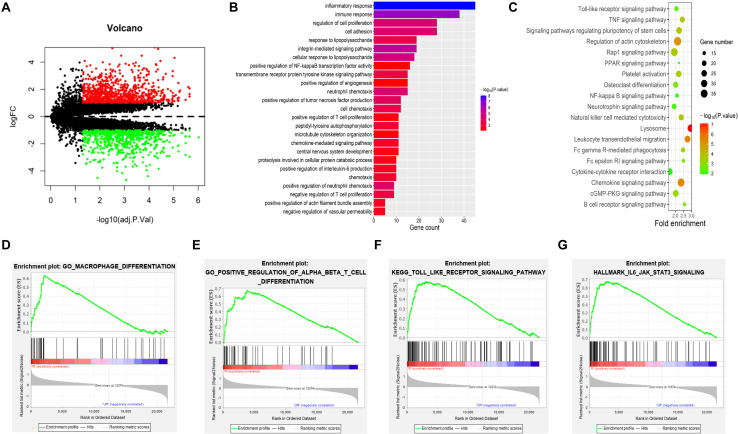
DEG and functional enrichment analyses. **(A)** Volcano plot of DEGs in IAs [*p* < 0.05 and log2| (fold change)| > 1] in the GEO IAs transcriptional profiles. There were 11 UIAs and 13 RIAs. **(B–G)** Visualization of the enrichment results. **(B)** GO terms are colored by the *p* value, and terms containing more genes tended to have a more significant *p* value. **(C)** KEGG pathway terms are colored by the *p* value, and terms containing more genes tended to have a larger size. **(D–G)** GSEA results show that macrophage differentiation, positive regulation of alpha beta T-cell differentiation, Toll-like receptor signaling pathway, and IL6–JAK–STAT3 pathway were enriched in RIAs compared with UIAs. DEGs, differentially expressed genes; GEO, Gene Expression Omnibus; IAs, intracranial aneurysms; UIAs, unruptured intracranial aneurysms; RIAs, ruptured intracranial aneurysms; GO, Gene Ontology; KEGG, Kyoto Encyclopedia of Genes and Genomes; GSEA, gene set enrichment analysis.

Details of the downloaded microarray data are provided in Table 1. In total, 1,471 DEGs were obtained, including 619 upregulated and 852 downregulated genes. The volcano plot of DEGs is shown in Figure 1A. GO enrichment analysis showed that DEGs were mainly enriched in the inflammatory response, immune response, cell proliferation, cell adhesion, angiogenesis, and neutrophil chemotaxis (Figure 1B). KEGG pathway enrichment analysis showed that the regulation of the actin cytoskeleton, leukocyte transendothelial migration, NF-κB signaling pathway, TLR signaling pathway, TNF signaling pathway, and chemokine signaling pathway plays important roles (Figure 1C). The GSEA results showed that the transcriptional profiles were enriched in macrophage differentiation, positive regulation of alpha beta T-cell differentiation, TLR signaling pathway and IL6–JAK–STAT3 pathway (Figures 1D–G).

**TABLE 1:** Summary of individual studies included.

Platforms	GEO accession	Sample size			Function	Contributors	Submission date
		
		UIAs	RIAs	Controls			
	GSE13353	8	11	0	DEG analysis and training set of IRMIR model construction	Kurki et al.	2008
GPL570	GSE26969	3	0	3		Li et al.	2011
	GSE6551	3	2	5		Tromp et al.	2006
GPL4133	GSE54083	5	8	10	Test set 1 of IRMIR model construction	Inoue et al.	2014
GPL6244	GSE15629	6	8	5	Test set 2 of IRMIR model construction	Pera et al.	2009

*A total of five microarray datasets retrieved from Gene Expression Omnibus (GEO) were included in this study. Three cohorts (GSE13353, GSE26969, and GSE6551) were selected for DEG analysis and used for the training set in the IRMIR model construction. Two cohorts (GSE54083 and GSE15629) were selected as test sets in the IRMIR model construction. Abbreviations: UIAs, unruptured intracranial aneurysms; RIAs, ruptured intracranial aneurysms.*

### PPI Network and Subnetwork Construction and Hub Gene Identification

**FIGURE 2:**
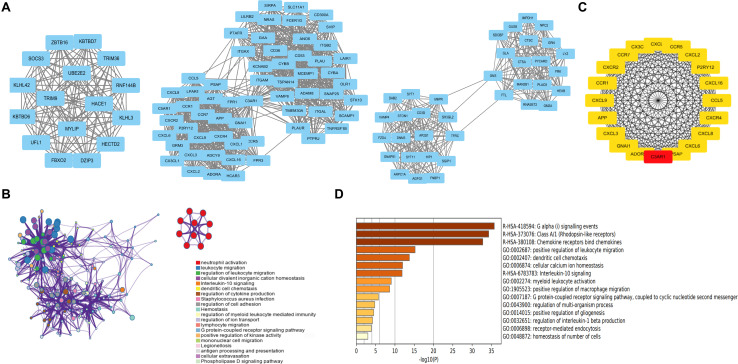
PPI network and subnetwork construction and hub gene identification. **(A)** Six subnetworks of PPI using MCODE plug-in of Cytoscape. The first three clusters are displayed. **(B)** The most related subnetworks were related to immune infiltration, neutrophil activation, leukocyte movement, and ubiquitination. **(C)** Twenty hub genes were identified by an MCC algorithm. **(D)** The hub genes were significantly enriched in positive regulation of leukocyte migration, dendritic cell chemotaxis, and positive regulation of macrophage migration. PPI, protein–protein interaction.

A PPI network of DEGs was constructed based on the STRING database. The network involved 964 nodes (DEGs) and 4,106 edges. In addition, the network was visualized using Cytoscape software. We obtained six subnetworks using the MCODE plug-in of Cytoscape. The first three clusters are shown (Figure 2A), and the most related subnetwork was related to immune cell infiltration, neutrophil activation, leukocyte movement, and ubiquitination (Figure 2B). Hub genes were identified by an MCC algorithm and included C3AR1, PSAP, CCL5, CCR5, CXCR2, CXCL8, CXCL1, CCR1, CXCL9, CXCR4, GNAI1, CXCL6, CXCL2, APP, CXCL3, CX3CL1, CCR7, ADORA3, P2RY12, and CXCL16 (Figure 2C). Among these genes, C3AR1, PSAP, CCL5, CCR5, CXCL8, CXCL1, CXCR4, APP, CX3CL1, and P2RY12 are reported to be related to IAs. Further functional enrichment analysis indicated that the hub genes were significantly enriched in the positive regulation of leukocyte migration, dendritic cell chemotaxis, and macrophage migration (Figure 2D).

### WGCNA of DEGs

**FIGURE 3:**
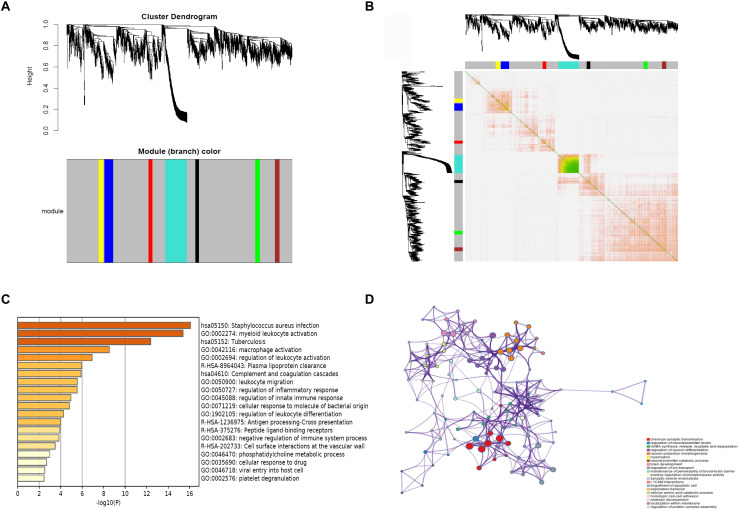
WGCNA of DEGs. **(A)** Cluster dendrogram and module assignment for modules from WGCNA. *X* axis represents genes, and *Y* axis represents height of the gene tree. The colored horizontal bar below the dendrogram represents the module that contains a group of highly connected genes. **(B)** Clustering of the DEGs by topological overlap shows that seven gene modules characterized by distinct expression patterns comprised highly coregulated genes. The color intensity in the heatmap is proportional to the interaction strength between two genes with green color for the strongest interaction and white color for no interaction. The seven modules are represented by the color bars along the *X* and *Y* axes. The blue and turquoise modules represent the top two modules. **(C)** The genes in the blue module are enriched in myeloid leukocyte activation, macrophage activation, and regulation of leukocyte activation. **(D)** The genes in the turquoise module are related to chemical synaptic transmission, regulation of neurotransmitter levels, and regulation of neuron differentiation. WGCNA, weighted gene coexpression network analysis.

WGCNA was performed, and seven modules were established, in which the blue and turquoise modules were the top two modules (Figures 3A,B). Functional enrichment analysis showed that the blue module was enriched in myeloid leukocyte activation, macrophage activation, and regulation of leukocyte activation (Figure 3C). The turquoise module was related to chemical synaptic transmission, regulation of neurotransmitter levels, and regulation of neuron differentiation (Figure 3D). The WGCNA results revealed that rupture of IAs was related to immune cell infiltration.

### Immune Cell Infiltration Analysis of RIAs and UIAs

**FIGURE 4:**
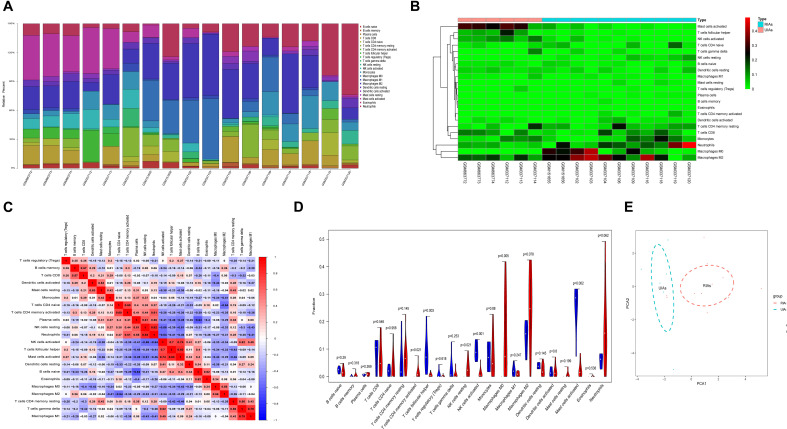
Immune cell infiltration analysis of RIAs and UIAs. **(A)** Histogram of the immune cell composition in different GSMs. **(B)** Heatmap of the immune cell content in different samples. **(C)** A correlation heatmap was generated to show the correlation of immune cells. **(D)** A violin map was graphed to show the difference of immune cells between the RIA group and UIA group. The blue column represents UIAs, and the red column represents RIAs. **(E)** PCA for immune cell infiltration between RIAs and UIAs. The blue circle represents UIAs, and the red circle represents RIAs. PCA, principal component analysis.

Because immune cell infiltration plays an important role in IA rupture, the immune cell infiltration pattern was determined using the CIBERSORT algorithm. The histogram in Figure 4A shows the immune cell composition in different samples. The neutrophils, monocytes, M0 macrophages, and M2 macrophages in RIAs increased significantly compared with those in UIAs. The mast cell content decreased significantly in RIAs (Figure 4B). Immune cell correlation analysis indicated that there was a significant positive correlation between follicular helper T cells and activated mast cells, as well as between resting natural killer (NK) cells and neutrophils. Plasma cells and naive B cells had a significant negative correlation (Figure 4C). In the violin chart, the Wilcoxon rank sum test was used to determine the significance of the differences and showed that activated CD4-positive memory T cells, resting NK cells, and M0 macrophages in RIAs increased significantly compared with those in UIAs. The contents of follicular helper T cells, activated NK cells, and mast cells in RIAs significantly decreased compared with the contents in UIAs (Figure 4D). The PCA results demonstrated that there was a difference in immune cell infiltration between RIAs and UIAs (Figure 4E).

### Construction of IRMIR

**FIGURE 5:**
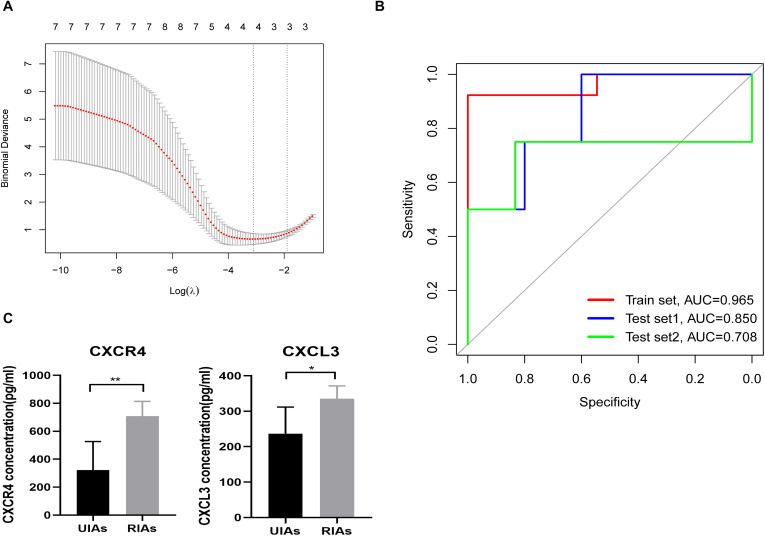
IRMIR construction and ELISA results. **(A)** The logistic regression model for the training set was conducted to identify an optimum linear combination in predicting responsiveness. **(B)** The AUC of the training set was 0.965. The AUC of test set 1 was 0.850, and the AUC of test set 2 was 0.708. The red line represents the training set. The blue line represents test set 1, and the green line represents test set 2. **(C)** CXCR4 and CXCL3 concentrations in the serum of RIA and UIA patients. The CXCR4 (*p* < 0.01) and CXCL3 (*p* < 0.05) concentration in the serum from RIA patients was significantly higher than that in UIA patients, respectively. IRMIR, immune-related risk model for IA rupture; ELISA, enzyme-linked immunosorbent assay. * represents *P* < 0.05, ** represents *P* < 0.01.

The expression data of the intersection of hub genes and immune-related genes, which contained 14 genes, were selected as the training set for IRMIR construction. The logistic regression model for the training set was conducted to find an optimum linear combination to predict responsiveness (Figure 5A). Then, ROC curve analysis of the training set and test set was conducted successfully in R. Risk scores were calculated for each sample as follows:

Risk score [IRMIR score] = 0.1035 × CXCR4 + 0.9049 × CXCL3 + –0.1150 × CX3CL1 + 0.6370 × CXCL16 – 7.2228

The area under the curve (AUC) of the training set was 0.965. The AUC of test set 1 was 0.850, and the AUC of test set 2 was 0.708 (Figure 5B).

### Enzyme-Linked Immunosorbent Assay Results

The CXCR4 concentration in the serum from RIA patients was 707 ± 47.26 pg/mL, which was significantly higher than that in UIA patients (322 ± 91.43 pg/mL, *p* < 0.01). Similarly, the CXCL3 concentration in the serum from RIA patients (335.2 ± 16.24 pg/mL) was also significantly higher than that in the serum from UIA patients (237.0 ± 33.54 pg/mL, *p* < 0.05) (Figure 5C).

### Construction of Immune-Related ceRNA Networks

**FIGURE 6:**
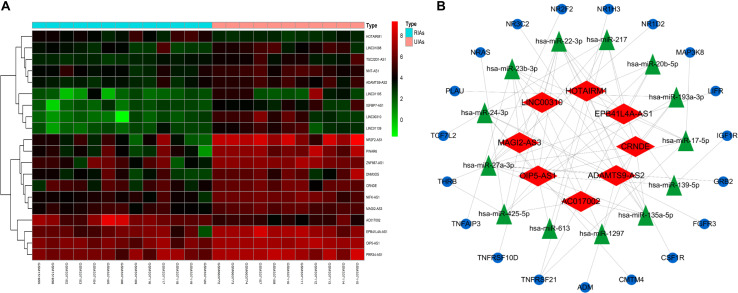
CeRNA network construction. **(A)** Differential expression heatmap of DElncRNAs. Blue represents RIAs, and orange represents UIAs. **(B)** CeRNA networks of UIAs and RIAs, which contain 8 DElncRNAs, 13 DEmiRNAs, and 19 DEmRNAs. The blue ellipse represents DEmRNAs, and the green triangle represents DEmiRNAs. The red diamond represents DElncRNAs. DEmRNAs, differentially expressed mRNAs; DEmiRNAs, differentially expressed microRNAs; DElncRNAs, differentially expressed lncRNAs.

To explore the internal molecular mechanisms, immune-related ceRNA networks were constructed. A total of 20 DElncRNAs were obtained, including 18 upregulated and 2 downregulated DElncRNAs. A differential expression heatmap of DElncRNAs is shown in Figure 6A. Thirteen DEmiRNAs were predicted to correspond to DElncRNAs, and 19 DEmRNAs were predicted as the target genes of the DEmiRNAs. In total, there were 8 DElncRNAs (EPB41L4A-AS1, AC017002, LINC00310, HOTAIRM1, MAGI2-AS3, ADAMTS9-AS2, CRNDE, and OIP5-AS1), 13 DEmiRNAs, and 19 DEmRNAs in the ceRNA networks. The resulting interactions were used to construct the lncRNA–miRNA–mRNA networks (Figure 6B).

## Discussion

The rupture of IAs can cause SAH, which has a critical impact on the health of patients (Abekura et al., 2020), causing a serious medical burden. Therefore, the timely identification of IAs with a higher risk of rupture and the implementation of intervention measures to prevent the progression from UIAs to RIAs are of great significance. Many studies have found that genetic factors play an important role in the rupture of UIAs (Bourcier et al., 2018; Bakker et al., 2020; Yanagisawa et al., 2020). In this study, bioinformatics analysis was conducted to explore the underlying mechanisms based on transcriptional profiles. We found that the inflammatory response and immune cell infiltration had a causal relationship with the rupture of UIAs. Moreover, the IRMIR, containing CXCR4, CXCL3, CX3CL1, and CXCL16, showed great value for predicting UIA rupture. ELISA showed that the CXCR4 and CXCL3 concentrations in the serum from RIA patients were significantly higher than those in the serum from UIA patients. Thus, the ceRNA network may be involved in regulating immune cell infiltration in IAs. Inflammation and immune cell infiltration play important roles in the formation and rupture of IAs (Frösen et al., 2012; Furukawa et al., 2020). As the most common inflammatory cells in IA lesions, macrophages regulate the occurrence and development of IAs by various mechanisms (Nakaoka et al., 2014; Shimonaga et al., 2018). Infiltrated macrophages are polarized by different stimuli to develop into M1 and M2 macrophages (Yamashiro et al., 2019). Macrophages of various polarized phenotypes regulate the inflammatory response not only by releasing cytokines and regulating other immune cells but also by releasing different cytokines to act on the extracellular matrix remodeling process, which regulates the brain in the occurrence and development of IAs (Hasan et al., 2012; Shao et al., 2017; Nowicki et al., 2018). Similar results were obtained from our study, which showed that M0 and M2 macrophages of RIAs increased significantly compared to those of UIAs. Moreover, the PCA results demonstrated that immune cell infiltration may be used to distinguish IAs with a high risk of rupture.

We constructed an IRMIR of IAs related to immune genes to predict the rupture risks of UIAs. More importantly, the model indicated that CXCR4, CXCL3, CX3CL1, and CXCL16 may be candidate biomarkers for predicting the rupture risks of UIAs. Recent findings have revealed that CXCR4 is associated with angiogenesis, inflammatory cell migration, and proliferation in IA walls (Hoh et al., 2014) and that it may play an important role in inflammatory activity in the abdominal aortic aneurysm (AAA) wall (Tanios et al., 2015). Moreover, high expression of CX3CL1 contributes to inflammatory infiltration into AAA tissue (Patel et al., 2008).

Intriguingly, the candidate biomarkers obtained from established IRMIR model are all secreted proteins, which can be detected in the blood. As the analysis of bioinformatics indicated that IA rupture was affected by different immune cell infiltration states, we hypothesized that the quantification of the candidate biomarkers in blood could partially reflect the risk of IA rupture.

Serum samples from RIA and UIA patients were collected to measure the concentrations of the candidate biomarkers. The result of ELISA showed that the CXCR4 and CXCL3 concentrations in the serum from RIA patients were significantly higher than that from UIA patients, indicating that CXCR4 and CXCL3 could be promising biomarkers for IA rupture in blood. In turn, these findings verified the accuracy of our model.

Although inflammatory macrophages infiltrate the aneurysm wall, a complete understanding of the relationship between their existence and the growth and rupture of IAs remains unknown. A previous study has shown that TNF-α secreted by macrophages is highly expressed in ruptured human aneurysms (Starke et al., 2014). Higher TNF-α expression is associated with increased expression of intracellular calcium ions (Ca^2+^) and intracellular calcium release channels (Jayaraman et al., 2008), which are related to the NF-κB signaling pathway and TLR signaling pathway. Moreover, the TLR4 pathway has been found to promote the development of IA rupture by accelerating inflammation in IA walls (Mitsui et al., 2020).

LncRNAs are sequences of more than 200 nucleotides in length (Batista and Chang, 2013) and have direct and indirect regulatory roles in tumors, cardiovascular diseases, and other diseases (Cremer et al., 2019). In this research, we performed an in-depth study on the molecular mechanism of immune cell infiltration. We obtained ceRNA networks with 8 DElncRNAs, 13 DEmiRNAs, and 19 DEmRNAs. CRANDE plays a role in tumor proliferation, migration, and the occurrence of many other diseases (Zhou et al., 2019a; Xie et al., 2020). CRANDE interacts with TLR3, and both correlate with advanced disease inflammation in sepsis patients (Yang et al., 2020). The other lncRNA, OIP5-AS1, has been reported to contribute to the progression of the atherosclerosis NF-κB pathway (Ren et al., 2020). Among the corresponding miRNAs, miRNA-23b-3p, miRNA-193a-3p, and miRNA-24-3p have reduced expression in human aneurysms (Jiang et al., 2013; Černá et al., 2019). Moreover, miRNA-17-5p, miRNA-20b-5p, miRNA-425-5p, miRNA-27a-3p, miRNA-22-3p, miRNA-135a-5p, and miRNA-139-5p have been reported to be related to inflammation and immune cell infiltration (Zou et al., 2016; Wang et al., 2020; Zhang Z. et al., 2020). MiRNA-217 and miRNA-613 have been found to be related to the NF-κB pathway in previous studies (Zhang L. et al., 2020). Among the target genes, LIFR, PLAU, ADM, MAP3K8, and TNFAIP3 may be involved in the formation of IAs, whereas LIFR has been reported to be associated with the occurrence and development of IAs (Yu et al., 2017). Moreover, a previous study has found that the inhibition of PLAU may protect against aneurysm formation (Rein et al., 2011). A prospective longitudinal cohort study has found that ADM is a marker of the risk of incident AAA (Acosta et al., 2018). In addition, MAP3K8 promotes inflammatory responses by acting as the essential kinase that propels NF-κB cascades (Hedl and Abraham, 2016; Dodhiawala et al., 2020). TNFAIP3 works as a negative NF-κB regulator, and its downregulation results in increased proinflammatory cytokine expression and aortic macrophage recruitment (Sudhahar et al., 2019), which may have a similar effect on the formation and rupture of IAs.

Our study performed integrated transcriptional profiling analysis and constructed an IRMIR. In detail, macrophage infiltration in RIAs was verified using a bioinformatics approach. The IRMIR built to predict the rupture risks of UIAs demonstrated a reasonably high predictive value, and ELISA was used to verify it. Furthermore, ceRNA networks were constructed to explore the potential mechanism of immune cell infiltration. However, because of the limited microarray data, the number of IA specimens for sequencing needs to be increased, and *in vivo* model validation may be a future research direction.

## Data Availability Statement

The raw data supporting the conclusions of this article will be made available by the authors, without undue reservation.

## Ethics Statement

The studies involving human participants were reviewed and approved by the Research Ethics Committee of Qilu Hospital of Shandong University. The patients/participants provided their written informed consent to participate in this study.

## Author Contributions

DS and XG designed the study and wrote the manuscript. GY and ZH helped with the study design. RZ and HX revised the statistical methodology. GL had primary responsibility for the final content. All authors have read, critically revised, and approved the final submitted manuscript, and all authors have agreed to be accountable for the content of the work.

## Conflict of Interest

The authors declare that the research was conducted in the absence of any commercial or financial relationships that could be construed as a potential conflict of interest.

## Footnotes

1 http://www.ncbi.nlm.nih.gov/geo/

2 http://www.perl.org

3 http://www.ensembl.org/index.html

4 http://www.r-project.org/

5 https://david.ncifcrf.gov/

6 http://www.broadinstitute.org/gsea

7 https://string-db.org/

8 https://www.immport.org/
